# Soil Contamination and Infection of School Children by Soil-Transmitted Helminths and Associated Factors at Kola Diba Primary School, Northwest Ethiopia: An Institution-Based Cross-Sectional Study

**DOI:** 10.1155/2022/4561561

**Published:** 2022-08-03

**Authors:** Aschalew Hussein, Megbaru Alemu, Animen Ayehu

**Affiliations:** ^1^Delgi Primary Hospital, Amhara National Regional Health Bureau, Amhara, Ethiopia; ^2^Department of Medical Laboratory Science, School of Health Sciences, College of Medicine and Health Sciences, Bahir Dar University, Bahir Dar, Ethiopia

## Abstract

**Background:**

Soil-transmitted helminths (STHs) are among the most prevalent parasitic worms infecting humans worldwide. They are still a major public health concern in the developing world, school-age children being the most affected segment of the population. Soil polluted with parasite ova and/or infective larvae is a source of human parasitic infections. There is a substantial shift in the notion of sanitation in many countries, and control of STHs contamination in the environment is becoming an emerging topic of research. However, data are scarce on the extent of soil contamination with STHs in Ethiopia in general and the study area in particular.

**Objective:**

This study aimed to determine the prevalence of STHs in soil, and school children and associated factors at Kola Diba primary school, Northwest Ethiopia.

**Methods:**

A school-based cross-sectional study was conducted in 2020 at Kola Diba primary school. A systematic random sampling technique was used to select 400 participants. About 150 soil samples were collected. A structured Amharic version questionnaire was used to collect data on sociodemographic variables and the factors associated with STH infection. Two grams of stool specimen was processed using the Kato-Katz technique. Data were entered and analyzed using STATA version 14.1. Binary and multiple logistic regressions were performed, and *P* < 0.05 was considered statistically significant.

**Result:**

The overall prevalence of soil-transmitted helminths was 50.0% with *Ascaris lumbricoides* (26.2%), hookworm species (16.5%), and *Trichuris trichiura* (3.5%). The overall soil contamination rate was 13.3% with *A. lumbricoides* (9.3%) and *T. trichiura* (4.0%). No habit of handwashing after toilet (AOR; 2.2, 95%CI: 1.3–3.6, *P* value = 0.002), no habit of regular shoe-wearing (AOR; 3.7, 95%CI: 2.1–6.2, *P* value <0.001), untrimmed fingernail status (AOR; 4.3, 95%CI: 2.6–7.1, *P* value <0.001), and playing with soil (AOR; 3.5, 95%CI: 2.2–5.7, *P* value <0.001) were significantly associated with STHs infection.

**Conclusion:**

The prevalence of STHs remains high among primary school children, with a considerable soil contamination rate. No habit of handwashing after defecation, untrimmed fingernail status, and no habit of regular shoe-wearing and playing with soil were significantly associated with the STHs infections.

## 1. Background

Soil-transmitted helminths (STHs) encompass three nematode species, namely *Ascaris lumbricoides (A. lumbricoides), Trichuris trichiura (T. trichiura),* and hookworms (*Ancylostoma duodenale*(A. duodenale) and *Necator americanus (N. americanus*)) [1]. Globally, 2 billion people are estimated to be infected with at least one species of STHs [2]. *Ascaris lumbricoides* is the most prevalent STH infecting about 1.2 billion people followed by *Trichuris trichiura* (infecting about 795 million people) and hookworms , infect nearly 740 million people worldwide [3]. Approximately 5.2 million disability-adjusted life years (DALYs) globally are lost due to STH infections [4]. Soil-transmitted helminthiasis affects mainly school children and they are the major cause of reduced physical and intellectual development, and impaired cognitive function and learning ability [5].

In Ethiopia, an estimated 81 million people comprised of 9.1 million preschool-aged children, 25.3 million school-aged children, and 44.6 million are living in areas endemic to soil-transmitted helminths [6]. The country harbors the 2^nd^ and 3^rd^ highest burden of ascariasis and ancylostomiasis, respectively. Moreover, 1/8^th^ of the Ethiopian population is believed to be infected with *T. trichiura* [7]. *Ascaris lumbricoides* and *T. trichiura* are transmitted through the fecal-oral route from contaminated water, hands, food, soil, and fomites. Hookworm species on the other hand are transmitted percutaneously, via active penetration of the skin by the filariform larvae. Moreover, ingestion of the filariform larvae can transmit *A. duodenale* [5].

Soil polluted with parasite ova, infective larvae, cysts, and oocysts can be a potential source of human parasitic infections [8]. In the last years, there has been a substantial shift in the notion of sanitation in many countries that control of STH contamination in the environment is becoming an emerging topic of research [9, 10]. For soil-transmitted helminths, the soil is the primary environmental reservoir for the eggs to get embryonated before transmission. In line with this, to become infective, the eggs of *A. lumbricoides* and *T. trichiura* must incubate at 5°C to 38°C for 8 to 37 days and 20 to 100 days, respectively. Similarly, the hatching of rhabditiform larvae from the eggs and then transformation into infective filariform larvae take 2 to 14 days of incubation at temperatures less than 40°C for hookworms [5].

Poverty, poor living conditions, personal and environmental hygiene, sanitation and water supply facilities, and open defecation are some of the contributing factors to the high prevalence of soil-transmitted helminths [11]. Place of defecation, source of dirking water, frequency of biting nails, absence of household latrine, rural residence, open defecation, illiteracy mother, and untrimmed fingernails showed a significant association with STHs infection [12–14].

Despite a range of prevention and control efforts, the prevalence of STHs is still high in several localities of Ethiopia. There is also a scarcity of data on environmental contamination by helminth eggs in the era of ongoing mass drug administration. In this regard, identifying hotspots of STH egg contamination in the domestic environment would be valuable for the design and evaluation of interventions aimed to reduce the transmission of STHs. In light of this, we aimed to assess soil contamination and prevalence of STHs among school children at Kola Diba primary school, Kola Diba District, Northwest Ethiopia.

## 2. Materials and Methods

### 2.1. Study Area

The district is located 780 kilometers far from Addis Ababa. The area comprises plateaus, plain, and rugged land with an altitude ranging from 1750 to 2100 meters above sea level. The area receives a mean annual rainfall and temperature of 1200 mm and 22°C. Vertisol and sandy soil account respectively for 80% and 20% of the soil type of district. A school-based cross-sectional study was conducted on school children and soil samples at Kola Diba primary school, East Dembia district, northwest Ethiopia.

### 2.2. Study Design and Period

A school-based cross-sectional study was conducted among school children and on soil samples at Kola Diba primary school, East Dembia district, northwest Ethiopia, from October 2020 to June 2021.

### 2.3. Source Population

All school children aged 6 to14 years at Kola Diba primary school were the source population of this study.

### 2.4. Study Population

All systematically selected Kola Diba primary school children were study population.

### 2.5. Study Participants

School children who fulfill inclusion criteria (aged between 6 and 14 years and can give stool samples) were considered study participants.

### 2.6. Exclusion Criteria

Children who took any anthelminthic drug within three months before the data collection period were excluded.

## 3. Study Size Calculation and Sampling Technique

### 3.1. Sample Size Calculation

The sample size was calculated using the single population proportion formula (*n*=*Z*_*a*/2_^2^*p*(1 − *P*)/*d*2), by taking *p*=54.9% from a previous study [15]. By adding a 10% nonresponse rate, a study size of 418 was calculated.

### 3.2. Sampling Technique

During sampling, the school children were stratified according to their educational level from grades 1 to 7. Allocation of the students to grade levels was performed proportionally to the number of students in each grade. Finally, the school children were selected using a systematic random sampling technique taking the class roster as the sampling frame. Moreover, a total of 150 soil samples were collected at a depth of 3 centimeters from the class entrance, school toilet entrance, inside the classroom, back of the class, play yards, and home entrance.

### 3.3. Data Collection and Processing

#### 3.3.1. Questionnaire Data

A pretested structured Amharic version questionnaire was used to collect data through a face-to-face interview technique.

### 3.4. Parasitological Survey

#### 3.4.1. Stool Sample Collection and Processing

Clean, dry, leak-proof, and labeled stool cups were provided to the study participants to collect about 2 grams of stool. The samples were transported to Dembia hospital laboratory and two Kato-Katz thick smears were prepared from each sample. In the Kato-Katz technique, approximately, 41.7-milligram feces was sieved through a 200 *μ*m Kato nylon screen mesh. The stool was transferred into a 6-millimeter hole of a template on a microscopic slide and covered with cellophane strips presoaked with 50% glycerol malachite green solution. The microscopic examination was performed within 30–60 minutes.

#### 3.4.2. Soil Sample Collection and Processing

All samples collected were placed in labeled plastic containers and were left air-dried overnight. The samples were examined using the sucrose flotation method, where soil samples were sieved through 150 *μ*m-mesh, and 2 grams of powdered soil was dispensed in test tubes mixed with 13 milliliters of distilled water. These tubes were centrifuged at 100 revolutions per minute (rpm) for 5 minutes. The supernatant was discarded and the sediment was homogenized with the same volume of distilled water and centrifuged at 800 rpm for 5 minutes and the supernatant was discarded again. Afterward, the tubes were filled with sucrose solution vigorously, stirred, and centrifuged at 100 rpm for 5 minutes. The supernatant was transferred to four other tubes until the fluid fill up to the brim of the tubes. Then, coverslips were put on top tubes and stayed for 10 minutes. The coverslips were removed and put on slides for microscopic observation at 10x and 40x objectives.

#### 3.4.3. Quality Assurance

To assure the reliability of the study findings expiry date of reagents was checked. All data collectors were trained. The principal investigator strictly followed the overall activities of the data collection daily.

### 3.5. Data Management and Analysis

All data were registered in the laboratory logbook and entered into Epidemiological Information statistical software (Epi Info) version 7.0 and finally transferred to STATA version 14.1 for analysis. Descriptive statistics were used to describe study participants and to compute the prevalence of parasites, while binary logistic regression was used to assess the association between explanatory variables and the outcome variable. Firstly, bivariate binary logistic regression analysis was done, and then to control the possible confounding, variables with a *P* value <0.25 were adjusted by multivariate logistic regression. *P* value <0.05 was considered a statistically significant association.

## 4. Results

In this study, a total of 400 school children were enrolled with a response rate of 95.7%. The mean (SD) age of the participants was 10.2 (±2.06) years. More than half (53.3%) of the study participants were males. Nearly half (48.5%) of the school children had illiterate parents (Table 1).

### 4.1. Prevalence of Soil-Transmitted Helminths

The overall prevalence of STHs was 50.0% (200/400); *A. lumbricoides,* Hookworms, and *T. trichiura* accounted for 26.2%, 16.5%, and 3.8%, respectively. Of the total examined schoolchildren, 46.5% and 3.5% had a single and double infection (*A. lumbricoides* and *T. trichiura*), respectively. The prevalence of STHs was slightly higher among males (51.2%) and children aged 6–10 years (50.6%) (Table 2).

### 4.2. Factors Associated with Prevalence of Soil-Transmitted Helminths

Nearly half of STH-infected participants experienced regular handwashing before meals. More than half (58.1%) of STH-infected children did not practice handwashing after defecation. About 66.7% of the study participants, whose household drinking water was sourced from streams were infected with STHs (Table 3).

In the bivariate analysis, variables such as no habit of handwashing before meal (*P*=0.0277), no habit of handwashing after toilet (*P* < 0.001), irregular shoe-wearing (*P* < 0.001), untrimmed fingernails (*P* < 0.001), playing with soil (*P* < 0.001), water source (*P*=0.086), and unavailability of the latrine (*P*=0.016) were significantly associated with STH infection. Variables with a *P* value <0.25 were adjusted by multivariate logistic regression. Accordingly, no habit of handwashing after toilet (AOR = 2.2, 95%CI: 1.34–3.62, *P*=0.002), irregular shoe-wearing (AOR = 3.7, 95% CI 2.14–6.23, *P* < 0.001), untrimmed fingernails (AOR = 4.3; 95% CI: 2.59–7.08, *P* < 0.001), and playing with soil (AOR = 3.5; 95% CI: 2.2–5.67, *P* < 0.001) were found to be significant predictors of STHs infection (Table 3).

### 4.3. Soil Contamination by Soil-Transmitted Helminths

In this study, the overall soil contamination rate was 17.3% with *A. lumbricoides* (9.3%), *T. trichiura* (4%), and *Eimeria Oocyst* (4%). The entrance of the toilet was more contaminated with *A. lumbricoides* (9.3%) and *T. trichiura* (4%). The prevalence of STH was higher in soils collected from the school toilet entrance but the entrance of the classroom and inside the classrooms were not contaminated by STHs (Table 4).

## 5. Discussion

In the current study, the overall prevalence of STHs among school children was 50.0% (95% CI: 45.0–55.0%). It is in line with studies from Durbete Town, Northwestern Ethiopia (54.9%) [15], Yirgacheffee, South Ethiopia (54%) [13], and Jimma Town, Southwest Ethiopia (53.5%) [16]. However, it was lower than the study findings from Chuahit, Dembia district, Northwest Ethiopia (66.7%) [17], Chencha Town, Southern Ethiopia (81%) [18], Benin City, South-South, Nigeria (70.8%) [19] and Hondurance [20]. This might be due to the study period (in this study period, the awareness of the population might increase latrine construction to reduce open field defecation, periodic deworming, particularly of the school-aged children, and control and prevention activities improved as compared to the former studies), diverse climate condition, and environmental sanitation, behavioral and cultural practices of the population. Increased prevalence of STH was observed in this study compared with studies from Gurage Zone, South Central Ethiopia (9.5%) [14], Gorgora, Northwest Ethiopia (24%) [21], Southwest Shewa, Ethiopia (15.8%) [22] and Kakamega County, Western Kenya (44.05%) [23]. The reason behind this might be due to limited health education access, high family illiteracy, no handwashing habits, untrimmed fingernail, open defecation practices, poor personal and environmental sanitation, and an insufficient number of toilets facilities at school, which have been observed in the present study.

The most prevalent STH in the study area was *A. lumbricoides* (26.2%%), which was higher than 21.7% in Yirgacheffe, South Ethiopia [13], 19.2% in Gorgora, Northwest Ethiopia [21], 13.8% in Durbete Town. Northwestern Ethiopia [15], 14.3% in Ejaji town, Ethiopia [12], 3% in Gurage zone, South Central Ethiopia [14], 6.6% in Southwest, Nigeria [24] and 22.7% in southern Nairobi [25]. The reason might be poor hand washing habits, no health education, untrimmed fingernails, inadequate availability of toilets, and poor environmental hygiene practice in the study area. Nevertheless, the current *A. lumbricoides* prevalence was lower than 63.8% in Chencha town, southern Ethiopia [18], 39.3% in Jimma Town, [16], 39.8% in Chuahit town, Dembia district, Northwest Ethiopia: [17], 39.5% in Honduras [20] and 43.5% in West Kenya [23].

Hookworm species infection was the predominant parasite infection in the majority of studies in Southwest, Nigeria (72.4%) [26], Ejaji town west showa (17.9%) [12], Yirgacheffe, South Ethiopia (16.7%) [13], Durbete town, Northwestern Ethiopia (46.4%) [15], but stands the second of STH (16.5%) in the present study. This figure is consistent with 15.9% in Honduras [20], 14.3% in Jimma Town, Southwest Ethiopia [16] but higher than 2.2% in Chencha town, southern Ethiopia [18], 2.2% in Gorgora, Northwest Ethiopia [21], 4.9% in Chuahit, Dembia district, Northwest Ethiopia [17], 4.2% in Gurage zone, South Central Ethiopia [14], 0.27% in West Kenya [23] and 0.1% in southern Nairobi [25]. The reasons might be the habit of the study participants about shoe wearing, open field defecation practices, and poor personal and inadequate environmental hygiene.

The prevalence of *T. trichiura* in the present study was 3.8%, which was much lower than studies reported in Jimma Town, Southwest Ethiopia (46.4%) [16], and Honduras (66.9%) [20]. But this finding was higher than the studies reported from Durbate Town, Northwestern Ethiopia (2.3%) [15], Gorgora, Northwest Ethiopia (1.7%) [17], Gurage zone, South Central Ethiopia (0.5%) [14], and west Kenya (0.82%) [23]. The difference might be due to the parasites' eggs and larval stages the variation of the soil temperature has been described as a key factor for the high transmission and prevalence of these parasites in the area [13]. The current finding is in line with the finding from Chuahit, Dembia district, Northwest Ethiopia (6.1%) [17], and Yirgacheffee, South Ethiopia (7.2%) [13].

Prevalence of *A. lumbricoides* was the most abundant among pupils of 6–10 years of age which was 56% with other age categories showing a prevalence was 44%. This prevalence was much higher than the prevalence reported in the Guragie zone, South Central Ethiopia (3%) [14], Southwest Nigeria (5.3%) [26], and Ejaji, Southwest Ethiopia (16.5%) [12]. There were higher levels of *A. lumbricoides* mean intensity in age groups 6–10 years. Children older than 10 years had the least mean intensity of *A. lumbricoides*. These results compare with a study carried out in Chencha town, Southern Ethiopia where children aged 9 to 11 years had a higher percentage of *A. lumbricoides* infections than schoolchildren in the other age groups [18].

The different prevalence of STHs in the study is in agreement with previous studies from Durbete Town, Northwestern Ethiopia (13.8%) [15], although a high percentage prevalence was noted for *A. lumbricoides* infections (26.8%) in this study. The current study had a low prevalence of *T. trichiura* and *hookworm*, 15.5% and 6.75%, respectively. This is in line with the previous STH survey in these regions (Gorgora, Northwest Ethiopia), *A. lumbricoides* infections were the most abundant compared to *T. trichiura* and hookworms [17]. This study reveals that large numbers of school children were infected with *A. lumbricoides*. It might be due to heavy soil pollution and sustain the transmission cycle for a longer period. Co-infections of STHs were less common in the study area. Multiple STHs infections were not common in other studies as elsewhere in Yirgacheffee, South Ethiopia [13].

Different risk factors, which contributed to the increased prevalence of STH among school children, were assessed using multivariable logistic regression analysis., This study revealed that handwashing after defecation, untrimmed finger, wearing shoes, and playing with soil were found to be independently significant factors of STH prevalence among school children. For children whose fingernails were untrimmed, the odds of having STH were 4.3 times that of children with trimmed fingernails (AOR = 4.3; 95% Cl: 2.5, 7.08). This shows that having untrimmed fingernails has a strong relation with infection by STH in the current study compared to other studies conducted from Buatjira, South-Central, Ethiopia [27] and Southwest Shoa [28]. The possible reason behind that association could be untrimmed fingernails hide disease-causing infectious agents and also serve as a good mode of transmission. Weekly nail clipping was also found to decrease intestinal parasite re-infection rates by 49% and dirt under fingernails may harbor different stages of parasites, which can be ingested during nail-biting or thumb [16].

This study showed similarly that children who had not washed their hands after deification were more likely to have STH 2.2 times higher compared to those who had washed their hands after deification. This finding is supported by the study conducted in Chuahit, Dembia, Northwest Ethiopia [17]. This is because hand washing reduces the risk of fecal-oral transmission of these parasites [5].

Another risk factor that contributed to the high prevalence of STHs among school children in Kola Diba primary school was shoe-wearing frequency. Children who did not wear shoes were more likely to have STH was 3.7 times higher compared to those who wore shoes (AOR 3.65 95% Cl; 2.14, 6.23). This finding is consistent with studies conducted in Ejaji, Northwest Ethiopia [12], Durbete Town, Northwest Ethiopia [15], Southwest Nigeria [26], Chuahit, Dembia district, Northwest Ethiopia [17], Yirgacheffee, South Ethiopia [13], and Northwest Ethiopia [29]. This might be due to when an individual is being barefooted there might be penetration of the skin by different parasites like the larva of hookworms that makes the individual vulnerablefor parasites listed above and contaminated with fecal matter [5].

Paying with soil was also another significant factor that contributed to the increased prevalence of STH among school children at Kola Diba primary school; children who play with soil were more likely to have STH 3.5 times compared to those who do not play with soil. This shows that having played with soil has a strong relation with infection by STH in the current study which is consistent with the study conducted in Northwest Ethiopia [29]. The reason behind that association might be during playing with soil ova, some of the helminths may remain viable in the soil for a long period so there is a probability of exposure to infective stages of *A. lumbricoides.*

Different studies were conducted to assess the prevalence of STH in the soil. The magnitude of STH in the soil as well as the area more contaminated by the parasite also assessed in the current study. The overall prevalence of STHs in the soil was 17.33% (95% Cl: 12.03, 24.33%). This is lower than studies from Philippines (41.33%) [30], rural Kenya (26.8%) [31], Cape Coast, Ghana [32] (95%), and South Nigeria (25.8%) [19]. Similarly, *A. lumbricoides* is the predominant parasite detected in the soil of the current study (9.3%). This is lower than the prevalence reported in studies of the Philippines (39%) [30] and South Nigeria (60.7%) [19].

Different studies tried to show the difference in the contamination rate of soil in different areas. The most prevalent area in this study was the entrance of the toilet of the school with *A. lumbricoides* being the most predominant parasite. This similar study from Benin City and its suburb, South Nigeria primary school [19]). The differences between the contamination rate may be due to environmental factors such as types of soil, rainfall, topographic, temperature, climate, and the use of human feces as fertilizers [16].

In this study, *A. lumbricoides* (9.3%) stands as the first and *T. trichiura* (4%) was the second more prevalent geo-helminths. Compared to other STH high prevalence of *A. lumbricoides* eggs in soil samples of this study is similar to other observations reported in the Philippines (98%) [30], Cape Coast, Ghana (39%) [32], and South-South Nigeria (59.5%) [19]. This can be explained by the fact that eggs of *Ascaris* have an inner shell layer of lipoprotein nature which makes them very resistant to adverse environmental conditions and live longer than other helminthes ova. Additional reason is that *A. lumbricoides* eggs can resist in different harsh environmental conditions. It might also be due to the scatter of *A. lumbricoides* eggs in the environment as one female *A. lumbricoides* compared to other helminths lay large number of eggs greater than 200, 000 eggs/day [16]. In this study, lower contamination rate of soil with *T. trichiura* eggs was observed. This might be due to their minimal scatter in the environment as one female *T. trichiura* liberates compared to *A. lumbricoides* less numbers eggs 2000 egg per day and also due to simply destruct of embryonated eggs by desiccation.

## 6. Conclusion

A high prevalence of the STHs was identified among the school children in the present study area, *Ascaris lumbricoides* being the most common even though different strategies handled to its prevention. The prevalence of STH in the soil was high. No habit of handwashing after defecation, untrimmed fingernail, no regular shoe-wearing habit, and playing with soil were significantly associated with the STHs infections.

## Figures and Tables

**Table 1:** Sociodemographic characteristics of school children (*n* = 400) at Kola Diba primary school, Northwest Ethiopia.

Variables	Categories	Frequency	Percentage
Sex	Male	213	53.3
	Female	187	46.7
Age	6–10	222	55.5
	11–14	178	44.5
Grade	1–4	231	57.7
	5–7	169	42.3
Parent occupation	Farmer	91	22.7
	Merchant	115	28.8
	Government employ	150	37.5
	Others	44	11.0
Parent education	Unable to read and write	194	48.5
	Primary school	23	5.7
	High school	22	5.5
	College and above	161	40.3
Religion	Orthodox	357	89.3
	Muslim	43	10.7
Parent marital status	Married	336	84.0
	Divorced	55	13.7
	Widowed	9	2.3
Family size	<5	106	26.5
	≥5	294	73.5

**Table 2:** Prevalence of STHs across various demographic variables among school children (*n* = 400) at Kola Diba primary school, Northwest Ethiopia.

Variables	Categories	Total examined	STHs infection	
			Positive	Negative
Age	6–10	222	110 (49.5)	112 (50.5)
	11–14	178	90 (50.6)	88 (49.4)
Sex	Male	213	109 (51.2)	104 (48.8)
	Female	187	91 (48.6)	96 (51.4)
Grade	1–4	231	116 (50.2)	115 (49.8)
	5–7	169	84 (49.7)	85 (50.3)
Parent occupation	Farmer	91	50 (54.94)	41 (45.1)
	Merchant	115	55 (47.8)	60 (52.2)
	Government employ	150	72 (48.0)	78 (52.0)
	Others	44	23 (52.3)	21 (47.7)
Religion	Orthodox	194	181 (50.7)	176 (49.3)
	Muslim	23	19 (44.2)	24 (55.8)
Parent education	Unable to read and write	22	85 (43.8)	109 (56.2)
	Primary and high school	161	18 (45.5)	27 (54.5)
	College and above	357	89 (55.3)	72 (44.7)
Marital status	Married	43	167 (49.7)	169 (50.3)
	Divorced	336	29 (52.7)	26 (47.3)
	Widowed	55	4 (44.4)	5 (55.6)
Family size	<5	9	144 (72.0)	56 (28.0)
	≥5	106	150 (75.0)	50 (25.0)

**Table 3:** Bivariable and multivariable analysis of factors associated with STH infection among Kola Diba primary school children (*n* = 400), Northwest Ethiopia.

Variables	Categories	Frequency	STH-infection N (%)	Statistics			
				COR (95% CI)	*P* value	AOR (95% CI)	*P* value
Hand wash before meals	Yes	347	176 (50.7)	1		1	
	No	53	24 (45.3)	1.6 (1.01, 2.6)	0.027	1.1 (0.6, 2.2)	0.77
Hand wash after toilet	Yes	178	71 (39.9)	1		1.00	
	No	222	129 (58.1)	1.4 (1.1, 1.8)	<0.001	2.2 (1.3, 3.6)	0.002
Regular shoe-wearing	Yes	249	95 (38.2)	1		1	
	No	150	104 (69.3)	2.3 (1.6, 3.2)	<0.001	3.7 (2.1, 6.2)	<0.001
Source of drinking water	Pipe	378	185 (48.9)	1		1	
	Stream	22	14 (66.7)	2.1 (0.9, 5.3)	0.086	1.3 (0.4, 4.3)	0.68
Fingernail status	Trimmed	148	36 (24.3)	1		1	
	Untrimmed	252	164 (65.1)	1.9 (1.4, 2.4)	<0.001	4.3 (2.6, 7.1)	<0.001
Washing vegetables	Yes	389	193 (49.6)	1			
	No	11	7 (63.3)	1.8 (0.51, 6.2)	0.37		
Playing with soil	Yes	214	175 (80.6)	1.9 (1.4, 2.5)	<0.001	3.5 (2.2, 5.7)	<0.001
	No	186	25 (13.7)	1		1	
Availability of latrine	Yes	314	147 (46.8)	1		1	
	No	86	53 (54.1)	1.61 (1.04, 2.5)	0.016	1.4 (0.8, 2.6)	0.25

**Table 4:** Parasitic contamination of soil (*n* = 150) at Kola Diba primary school, Northwest Ethiopia.

Helminth Species	Site of soil sample collection						
	Class entrance (*n* = 25)	School toilet entrance (*n* = 25)	Inside the classroom (*n* = 25)	Back of the classroom (*n* = 25)	Play-yard (*n* = 25)	Home entrance (*n* = 25)	Total Pos (%)
*A. lumbricoides*	0	4 (2.7%)	0	3 (2%)	1 (0.6%)	6 (4%)	14 (9.3)
*T. trichiura*	0	2 (1.3)	0	2 (1.3%)	0	2 (1.3)	6 (4.0)
Eimeria oocyst	0	0	0	0	0	6 (4%)	6 (4.0)
Total	0	6 (4%)	0	5 (3.3%)	1 (0.6%)	14 (9.3%)	26 (17.3)

Notice: Pos = positive; *n* = number of soil samples taken at each site

## Data Availability

The datasets analyzed during the current study are not publicly available due to institutional regulation but are available from the corresponding author on reasonable request.

## Abbreviations

AOR: Adjusted odds ratio
CI: Confidence interval
COR: Crude odds ratio
EPG: Eggs per gram
FEC: Formol-ether concentration
KK: Kato-Katz
SPSS: Statistical Package for the Social Sciences
SSA: Sub-Saharan Africa
STH: Soil-transmitted helminth
WHO: World Health Organization.
